# Subserosal Supernumerary Muscle Layer of the Intestine: A Peculiar Bowel

**DOI:** 10.7759/cureus.60096

**Published:** 2024-05-11

**Authors:** Mehul Gupta, Arun Kumar, Vitish Singla, Sandeep Aggarwal, Prasenjit Das

**Affiliations:** 1 Department of Surgery, All India Institute of Medical Sciences, New Delhi, New Delhi, IND; 2 Department of Pathology, All India Institute of Medical Sciences, New Delhi, New Delhi, IND

**Keywords:** muscle abnormality, bowel obstruction, bowel histology, supernumerary muscle layer, gut muscle

## Abstract

The presence of a supernumerary subserosal muscle layer of the bowel is an extremely unusual congenital development. The following is a report of diffuse involvement of the intestine with a supernumerary subserosal muscle coat. The current patient, a 29-year-old male, was evaluated in January 2022 for a long-standing history of subacute intestinal obstruction (SAIO). A preoperative CT scan of the abdomen and pelvis suggested mild dilatation and clumping of ileal loops in the right iliac fossa, with a subtle wall thickening of up to 5 mm. Intraoperatively, dense adhesions were noted between clumped bowel loops and the anterior abdominal wall. Following adhesiolysis, ileocecal resection with ileocolic anastomosis was done. The histopathological examination of the resected bowel segment showed irregular hypertrophy of circular and longitudinal muscle layers with the presence of an additional smooth muscle coat outer to the outer longitudinal layer that was seen in the ileum as well as the appendix. No evidence of vacuolar degeneration was noted, and ganglion cells were seen to be adequately present. The presence of additional smooth muscle bundles in the subserosa was confirmed with positive actin immunostaining. Additionally, CD117 staining was done that revealed a normal network of interstitial cells of Cajal. No evidence of active inflammation was noted in the resected bowel segment. Findings from the current case bring to light an extremely rare malformation of the muscularis propria of the intestine, namely a supernumerary subserosal muscle coat.

## Introduction

Intestinal obstruction remains one of the most common entities encountered in surgical practice [1]. While acute obstruction usually presents with prominent diagnostic clues (such as severe colicky pain with no passage of stools/flatus and recurrent emesis), subacute intestinal obstruction (SAIO) may have a variable presentation with patients presenting only with intermittent obstructive symptoms of varying severity [2]. The commonly stated reasons for the development of SAIO are adhesions from prior surgeries, neoplasms, obstructing luminal growths, Crohn’s, and intestinal herniation [3]. Soyer et al. (2015) [4] reported for the first time the presence of a supernumerary subserosal muscle coat in a three-day-old neonate with segmental ileal dilatation who presented with obstructive symptoms, namely abdominal distension and bilious vomiting. A brief review of the literature using the search term (((Supernumerary) AND (Muscle)) AND ((Bowel) OR (Intestine))) yielded no further reports on similar findings in resected bowel specimens. To the best of our knowledge, this is the first case reporting diffuse involvement of an adult intestine with a supernumerary subserosal muscle coat layer.

## Case presentation

A 29-year-old male presented to the Surgical Clinic of our tertiary care teaching hospital in January 2022, with a long-standing history of recurrent colicky abdominal pain occurring two to three times every month. There were no associated complaints of fever/recurrent emesis/absolute obstruction to the passage of stools or flatus. In 2014, the patient was started on empirical anti-tubercular therapy (ATT) for 12 months in view of a radiological suspicion of abdominal tuberculosis but did not note any improvement in symptoms. Anti-tubercular therapy was again repeated in 2017 following consultation from a different surgical team but to no avail. The patient had undergone an enterostomy in early childhood in view of severe worm infestation, and an open appendicectomy in 2016 with primary repair of an iatrogenic ileal injury. He had no chronic co-morbidities. At the time of presentation to the clinic, the patient was hemodynamically stable. The per-abdominal examination revealed a soft, non-distended, non-tender abdomen with a scar mark in the right iliac fossa suggestive of a prior surgical procedure.

The patient was taken up for a contrast-enhanced CT scan of the abdomen and pelvis to look for the cause of his intermittent bowel obstruction. Mild dilatation and clumping of ileal loops were seen in the right iliac fossa with a subtle wall thickening of up to 5 mm. The rest of the bowel loops were noted to be normal. No particular transition point could be identified between the dilated and non-dilated bowel loops. He was then prepared for elective diagnostic laparoscopy and was taken for surgery on February 4, 2022. Intraoperatively, clumping of terminal ileal loops with dense adhesions between the clumped bowel loops and anterior abdominal wall was noted (see Appendix 1). In view of inadvertent iatrogenic injury to the ileal loops during adhesiolysis, ileocecal resection involving 160 cm of distal ileum and the cecum (see Appendix 2) with ileocolic anastomosis of the remnant healthy bowel was done for the patient, and the resected bowel specimen was sent for pathological evaluation.

**Figure 1:**
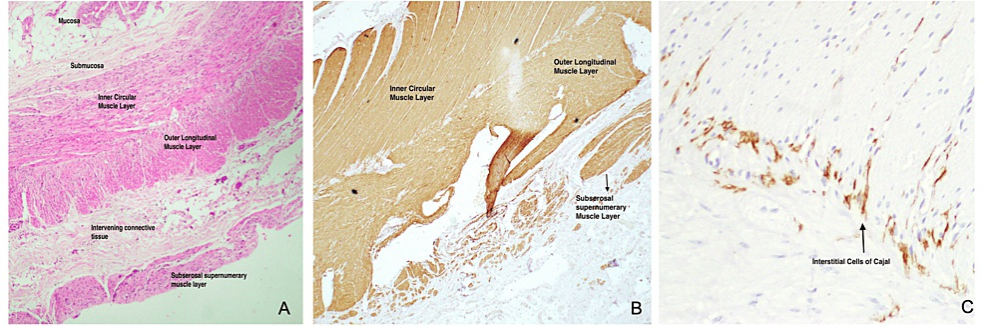
Histopathological specimen of the resected bowel wall A: H&E staining of bowel wall from resected specimen; B: Smooth muscle actin (SMA) stain showing the presence of subserosal supernumerary muscle layer; C: CD117 staining of interstitial cells of Cajal (ICC) showing the presence of ICC predominantly between inner circular and outer longitudinal muscle layers

The postoperative course for this patient remained largely asymptomatic. The patient was eventually discharged on postoperative day eight (POD-8), in a hemodynamically stable state with complete tolerance for oral diet. The histopathological (HPE) report of the resected bowel segment showed irregular hypertrophy of the circular and longitudinal muscle coats with the presence of an additional smooth muscle layer outer to the outer longitudinal muscle coat, throughout the resected specimen (Figure 1).

No evidence of vacuolar degeneration was noted, and ganglion cells were seen to be adequately present. The presence of additional smooth muscle bundles in the subserosa was confirmed with positive actin immunostaining. Additionally, CD117 staining was done which revealed a normal network of interstitial cells of Cajal (ICC), with no extension of the ICC to the supernumerary muscle layer. No evidence of active inflammation was noted in the resected bowel segment. The extra layer of smooth muscle was also seen to be present in sections examined from the appendicular stump.

**Figure 2:**
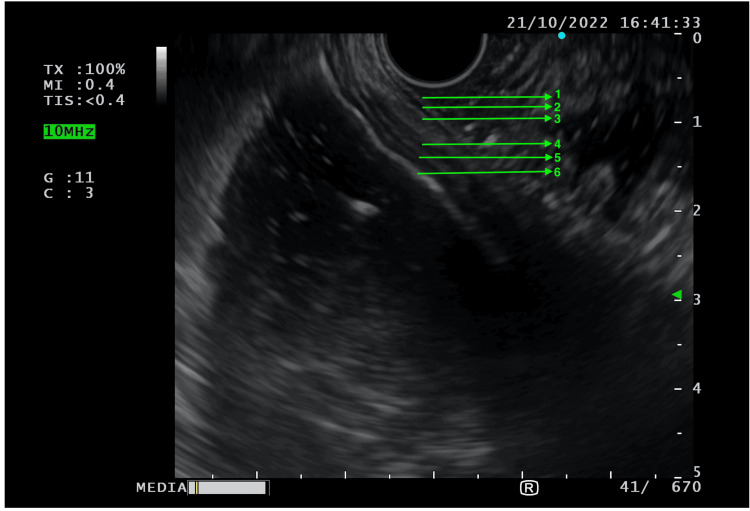
Endoscopic ultrasound (EUS) findings of distal duodenum and jejunum, showing supernumerary subserosal muscle layer 1: Mucosa, 2: Muscularis mucosa, 3: Submucosa, 4: Muscularis externa, 5: Subserosal supernumerary muscle layer, 6: Serosa

The patient was reviewed at eight months following primary surgery. He reported a persistent mild generalized abdominal discomfort following meals that resolved spontaneously. No episodes of severe abdominal pain/vomiting/constipation were noted by the patient after being discharged following surgery in February 2022. In view of the unusual HPE findings, an endoscopic ultrasound (EUS) was done to assess the bowel wall in the distal duodenum (D3/D4) and proximal jejunum, which revealed an additional muscle layer beyond the muscularis propria (Figure 2).

## Discussion

A supernumerary subserosal muscle layer was first reported by Soyer et al. [4] in a three-day-old neonate who was diagnosed with segmental ileal dilatation following evaluation for obstructive symptoms. Similar to our case, Soyer et al. [4] noted the presence of additional muscle fibers beyond the outer longitudinal muscle layer. The histopathological analysis, in either case, yielded no evidence of vacuolar degeneration in the additional muscle layer, thus suggesting that such an anatomical bowel might have resulted from a developmental disorder instead of an inflammatory process. However, there are a few important differences between the findings previously reported by Soyer et al. [4] and the findings in the current report. Firstly, Soyer et al. [4] noted only a segmental distribution of the additional muscle coat, while EUS findings in the current case suggested the involvement of the duodenum as well as the proximal jejunum, hence indicating that the entire small bowel may have been involved by the supernumerary subserosal muscle coat that is described above. Secondly, they noted the presence of ICC on both sides of the longitudinal muscle layer. In contrast, ICC in our patient were seen only in their normal distribution (i.e., predominantly in the myenteric plexus) and were not seen to be associated with the additional muscle layer. This is important as ICC are responsible for generating electrical rhythmicity and for synchronizing the activity of smooth muscle cells into purposeful peristaltic contractions [5]. Intuitively, the absence of ICC in association with an additional muscle layer might predispose an individual to asynchronous peristaltic contractions.

The presence of an additional muscle coat has been noted previously in multiple studies evaluating pediatric patients with obstructive symptoms [6-8]. However, this has never been noted previously in a subserosal location and in association with minimal symptoms for the patient. Smith and Milla [6] reviewed histological variations of smooth muscles in resected bowel specimens in a pediatric population and noted additional muscle layers between the inner circular and the outer longitudinal muscle coat, or inner to the inner circular muscle coat. Supernumerary muscle coat has also been described previously in children with Mowat-Wilson syndrome commonly presenting with constipation; however, this additional layer has only been described in submucosal locations [7,8].

Due to its extremely rare occurrence, the symptomatology and implications of a diffuse subserosal supernumerary muscle coat layer remain unclear. Notably, the likely cause of severe right iliac fossa pain and obstructive symptoms in our patient was the presence of dense adhesions along the terminal ileal loops. The absence of ICC in the additional muscle coat was likely associated with a certain extent of asynchrony in peristaltic contractions of the intestine, thus yielding the persistent mild postprandial abdominal discomfort that this patient had been experiencing despite undergoing adequate adhesiolysis and jejunocolic anastomosis in the primary surgery. However, in view of the diffuse bowel wall involvement and low-grade symptoms, any form of additional active intervention was not warranted for this condition.

## Conclusions

Supernumerary muscle coats of the intestine are an extremely rare group of structural variants of the bowel wall. The current report is the first account of a diffuse subserosal supernumerary muscle coat layer, resulting in persistent mild postprandial abdominal discomfort in an adult male. The etiology and significance of a diffuse subserosal supernumerary muscle coat layer are yet unknown. However, the possibility of asynchronous peristaltic contractions due to the absence of ICC within the supernumerary layer may be of clinical importance, particularly in the setting of an unexplained persistent mild abdominal discomfort.

## Figures and Tables

**Figure 3:**
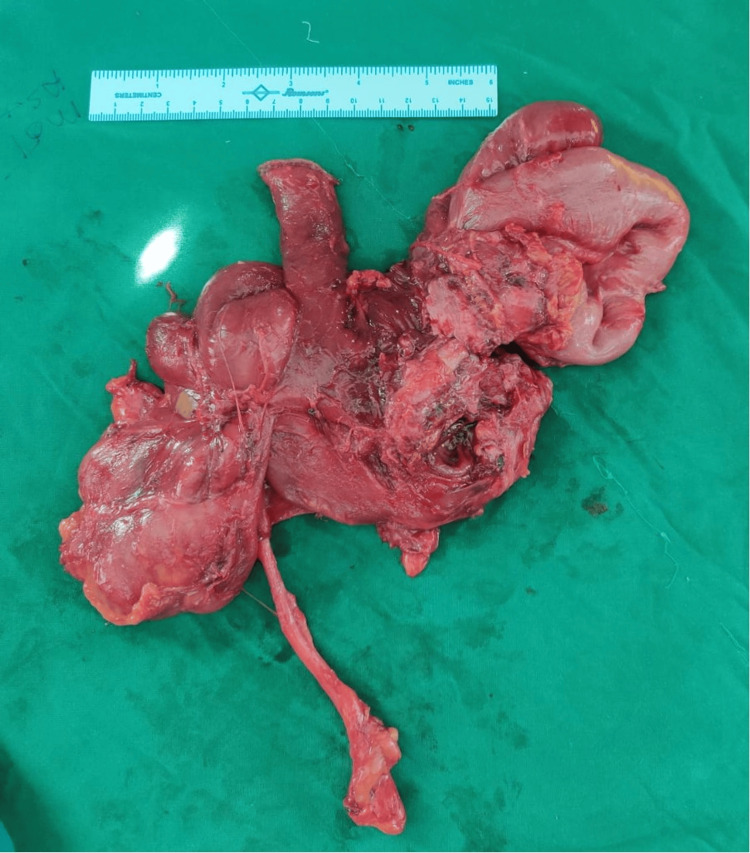
Resected ileocecal specimen

**Figure 4:**
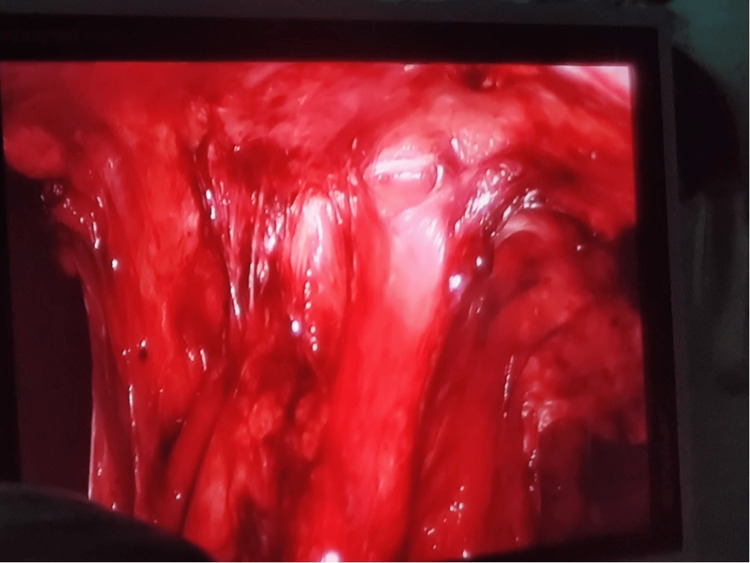
Intraoperative diagnostic laparoscopy showing dense adhesions

## Appendices

Appendix 1

Figure 3 shows the resected ileocecal specimen.

Appendix 2

Figure 4 shows intraoperative diagnostic laparoscopy showing dense adhesions.
